# A prospective clinical review of “multi model” approach for treating ear keloids

**DOI:** 10.4103/0970-0358.41103

**Published:** 2008

**Authors:** Ganesh K. Narakula, R. K. Shenoy

**Affiliations:** Consultant Plastic Surgeon and Associate lecturer; Queen Elizabeth Hospital and School of Clinical Medicine, University of West Indies, Barbados, West Indies; 1 Consultant Radiation Oncologist and Associate Lecturer; Queen Elizabeth Hospital and School of Clinical Medicine, University of West Indies, Barbados, West Indies

**Keywords:** Early recurring lesion, intralesional triamcinolone, keloid, radio therapy

## Abstract

This is a prospective clinical study of 46 ear keloids in 31 patients (with a mean follow-up of 18 months) treated from January 2006 to December 2006 at The Queen Elizabeth Public Hospital, Barbados, West Indies by a single surgeon. The mean age is 21.9 years (range 3-66 years). Seven out of 46 lesions were recurrent lesions following previous surgery. All the lesions were excised surgically (extralesional). Ten out of 31 patients were given postoperative, Intralesional Triamcinolone starting from the 1^st^ post operative visit on three visits at monthly intervals. Fourteen patients were given postoperative superficial X-ray therapy of 12 Gy in three equal fractions on three consecutive days starting from the 3^rd^ postoperative day. Seven recurrent keloids of this study were given a combination of both superficial X-ray therapy and intralesional triamcinolone. All patients were followed at monthly intervals for three visits from the time of surgery and every three months until the end of the 1^st^ year and then every six months thereafter. Five of 46 postoperative surgical wounds showed evidence of recurrence during the 1^st^ year but could be suppressed with Intralesional triamcinolone. This study confirms that surgical excision of keloids supplemented with radiotherapy and/Intralesional triamcinolone is a reliable method with few complications. In addition, the study concludes that the *key* in preventing recurrence is regular clinical follow-up to encounter early recurring lesion (clinical evidence of raised scars or palpable nodules if deep seated) which is 100% susceptible to Intralesional triamcinolone for 2-3 times at monthly intervals.

## INTRODUCTION

It is well known that keloids are “Confused scars that do not know when to stop growing”. The basic pathology is an imbalance between anabolic (proliferation) and catabolic (apoptotic) phases of the healing process.[1]

The various treatment modalities so far described in managing keloids are[2,3] surgical excision, intralesional steroidal injections, compression therapy with silicon sheets, cryotherapy, laser, α-2b interferon and chemotherapeutic agents like 5-fluorouracil. Surgical excision totally eliminates the lesion but the main disadvantage is ≥50% recurrence [3] if used alone. The disadvantage with the other procedures is incomplete ablation of the lesion leaving poor aesthetic results. To remove the lesions totally and to prevent recurrence needs “surgical excision in combination with one or more of the other modalities of treatment”. [2–7]

We are reporting the clinical outcome of 46 ear keloids in 31 patients treated from January 2006 to December 2006. The prime modality of treatment is “surgical excision” of lesions supplemented with radiotherapy and/intralesional triamcinolone. Three protocols were adopted.

## MATERIALS AND METHODS

This prospective study was done at The Queen Elizabeth Public Hospital, Barbados, West Indies, which is a referral hospital to 300,000 Islanders and for other English-speaking Eastern Caribbean people. Majority of the Islanders are ethnically of Black African- descent.

The sample consists of 46 ear keloids in 31 consecutive patients (all of them are Islanders). All patients in this series were treated by a single surgeon. The patients had ages ranging from 3-66 years, the mean age being 21.9 years. Of the 31 patients, 20 had their ears pierced between the ages of 12-18 years. Only four patients had their ears pierced before ten years of age. Except for one patient who was 63 years old, all others got their ears pierced before the age of 34 years.

Seven out of 31 patients were male, 24 were female, 15 patients had bilateral lesions. Out of 46 lesions on the ears, 33 were on the lobule and 13 were on the cartilaginous part.

Of the 46 lesions, only one was of posttraumatic origin, the rest were following ear piercing, seven were recurrent following earlier surgical excision (two of them recurred twice after subsequent surgical excisions). The recurrence of six out of these seven lesions was within a year from the time of surgery.

Two of the 31 patients had surgery simultaneously in other regions; one for two lesions on the left cheek and the other patient on the upper sternum.

The duration of the lesions varied from 1-15 years (average 3.8 years); summary is shown in Table 1.

**Table 1:** Analysis of sample and results

Total number	31 patients, 46 ears
Age	3 to 66 years (Mean age 21.9 years)
Sex	Male-7, Female-24
Unilateral *vs.* Bilateral	Unilateral-16, Bilateral-15
Recurrent lesions	7
Location	Lobule of ear-33, Cartilaginous pinna-13
Duration of keloids	1 to 15 years (Mean 3.8 years)
Etiology of lesions	Piercing-45 ears, Posttrauma-1ear
Age of piercing	<10 years-4 pts,
	12-18 years-20 pts
	19-34 years-6 pts
	63^rd^ year-1Pt
No of pts operated for keloids at other regions along with ear keloids	2
Treatment given	Surgery + Rtx-14 pts
	Surgery + Intralesional triamcinolone-10 pts
	Surgery + Rtx + Intralesional triamcinolone -7 pts
Evidence of recurrence(Early recurring lesion)	Surgery + Rtx-2 pts
	Surgery + Intralesional triamcinolone-1 pt
	Surgery + Rtx + Intralesional triamcinolone-2 pts

Diagnosis was done based on clinical criteria. Initially, two lesions were sent for histological examination but this practice was discontinued as literature reviews deemed histological examinations as being necessary only in cases of uncertainty.[2]

Identifying ‘Early recurring lesion’: The suture line was examined in good light for any raised scar and palpated for any palpable lesion during every visit.

### Protocols adopted

Before starting the treatment protocol, all patients were meticulously counseled[3] about the etio-pathogenesis of the keloids, the treatment plan and its duration, likely complications of all modalities of treatment options. They were warned not to plan pregnancy for a period of two years from the time of consultation and not to miss follow-up as well as of the probability of treatment failure. Contact details of every patient were recorded and all patients were advised to contact the surgeon for an early appointment if they found any recurrence (self identification of recurrence is not difficult; on most occasions, the patient walks into the clinic saying ‘Something is palpable’).

*1. Protocol-A:* Surgical excision + Radiotherapy After surgical excision of the lesion, radiotherapy was started on the 3^rd^ post operative day (ideally to be started on the 1^st^ post operative day but as I operated on these patients on Fridays, radiation could be started only on the following Monday) and radiation was delivered in three equal fractions on three consecutive days.

Follow-up was at monthly intervals for three visits followed by quarterly visits until the end of the 1^st^ year and biannual visits until the end of the 2^nd^ year.

Topical mometasone cream was prescribed at the first post operative visit and continued until pigmentation at the site of the radiation scar had cleared up.

Intralesional triamcinolone was reserved for those who showed clinical evidence of recurrence during the follow-up and continued for three injections at monthly intervals.

*2. Protocol-B:* This is the protocol used for those who were not given postoperative radiotherapy. After surgical excision of the lesion, the patient is followed similarly as in Protocol A. The patient was given Intralesional triamcinolone starting from the first post operative visit (a month from the time of surgery) and continued for three consecutive monthly doses.

In addition, Intralesional triamcinolone was reserved for any evidence of recurrence from either group throughout the period of follow-up and continued for three injections at monthly intervals.

*3. Protocol-C:* This is a combination of the above two protocols. This protocol is used for recurrent keloids only (surgery + Radiotherapy (RTx) + Intralesional triamcinolone)

### Details of treatment modalities used

*1. Surgical excision:* Is the prime modality of treatment for all cases. [2–5]

Except for two children, all the rest were operated under local anesthesia with epinephrine. All lesions were excised radically through normal-appearing tissue and meticulous haemostasis was secured. Few subcutaneous sutures were used to take away the tension using 5-0 Prolene. The same was used for skin closure. Patients were discharged after applying pressure dressing along with antibiotic and analgesic prescriptions. Sutures were removed on the 5^th^ postoperative day.

**Figure 1:**
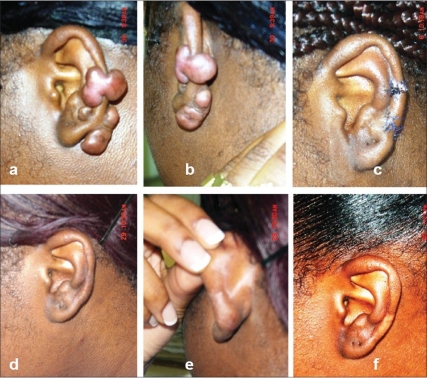
(A) 20 yrs old lady got piercing both pinnae 2 yrs ago (Similar but less severe lesions on Rt pinna also), Upper lesion is of Dumbell nature, better seen in 1-B, Lower lesion is on medial aspect only but involving Cartilage, (B) Posterior medial view, (C) Operated on 26/1/2006, Post op RTx given and followed by topical mometasone cream to clear up pigmentation; Upper lesion-Wedge resection and primary closure; Lower lesion-simple excision of lesion along with involved cartilage and primary closure, (D) 7 Months after Surgery, (E) Posterior medial view, (F) 22 Months after surgery, never showed any signs of recurrence
- Most of the lesions on the lobules (21/33) required simple excision and primary closure. Two lesions of <0.5 cm diameter on the cartilaginous pinna (lower lesion of Figure 1) could be managed without wedge excision.
- Four Keloids on the lobules of the ears situated on the posterior medial aspect (common location of lobule keloids in this study) with a broad base required closure using Limberg's rhomboid flap from the posterior auricular region.
- One very big lesion located subcutaneously on the cartilaginous ear (9) was debulked [Figure 2] as nothing else could be done.

- Excision of a wedge of the ear along with the lesion was required for 12/33 lesions of the lobule (lesions which were very big, lesions with broad bases, lesions located on the margin of the lobule and lesions with dumbbell extension to both the surfaces) and 10/13 lesions of the cartilaginous pinna (upper lesion of Figure 1).

**Figure 2:**
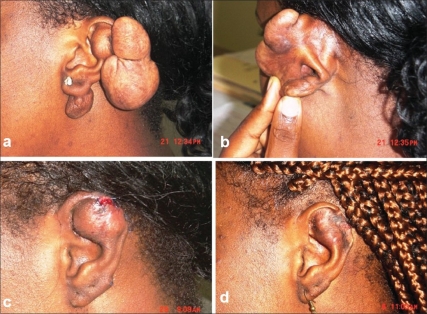
(A) 23 yrs old lady, H/o piercing at the age of 16 yrs, followed by keloid; Operated in 2002 with out post-op RTx and not followed regularly with treating surgeon. Recurred soon after. (B) Occupying most of the circumference of the helical rim and extensive subcutaneous extension on lateral aspect. Even though it occupies most of the circumference, pedunculated in nature. (C) Extensive sub cutaneous lesion on lateral aspect was debulked as nothing else could be done. This type of management is not possible with most of the other keloids (Compare with lesions of Fig. 1), which are true scars covered with epidermis only (Here the lesion is under normal appearing skin (Fig. 2-A). Keloid filletted flap? (Ref 9). (D) After debulking the patient in Fig. 2-C was followed with IL Triamcinolone Monthly for 4 times. this pictute is 11 Months after surgery

*2. Radiotherapy:* Superficial X ray therapy at 100 kev to a dose of 12 Gy in 3 fractions over 3 days was administered after obtaining informed consent to patients who were above 18 years of age. Twenty one out of 31 patients including seven with recurrent keloids were given radiotherapy. [2–7]

*3. Topical mometasone cream:* Patients who were given radiotherapy were prescribed a topical application of Mometasone cream over the scarred area starting from the first postoperative visit. It took about 4-8 Weeks to clear the pigmentation left by radiation.

*4. Intralesional triamcinolone:* Triamcinolone 40 mg/ml is given intralesionally to:

1. All patients who were not given radiotherapy after surgical excision,
2. Along with radiotherapy for recurrent keloids after surgical excision and
3. Those that showed evidence of recurrence during follow-up.

Intralesional steroid therapy was started during the first visit (one month from the time of surgery) and continued for 3-5 sessions at monthly intervals.[2,3,7]

The maximum dose used in adults was never more than 120 mg.

## RESULTS

All the patients were followed for an average period of 18 months from the time of surgery (longest follow-up being 23 months). Four out of 31 patients who missed follow-up were interviewed over the phone.

Protocol-A: Two postoperative wounds showed recurrence (clinical evidence of raised scar or palpable nodule if deep seated) at a point on the suture line where maximum tension is likely and were controlled totally with Intralesional triamcinolone monthly for 2-3 months.

Protocol-B: Evidence of recurrence during follow-up was noticed with one patient, who had mild postoperative infection. It was controlled well with three monthly injections of Intralesional triamcinolone.

Protocol-C: Two Patients showed recurrence and were controlled totally using Intralesional triamcinolone.

Surprisingly, two patients (both belonging to Protocol-A) who were operated for associated keloids along with ear keloids showed evidence of recurrence at a postoperative scar other than the ear and required Intralesional triamcinolone for controlling it, but not at the postoperative ear scar. The same is summarized in Table 1.

All 31 patients are free from recurrence now.

### Complications

All patients showed depigmentation and atrophy of dermis after intralesional triamcinolone injections. Depigmentation and dermal atrophy were eventually reverted after stopping intralesional triamcinolone with out any treatment. One patient had postoperative wound infection. Another patient had partial necrosis of Limberg's flap but the subsequent course was uneventful.

## DISCUSSION

Twenty seven out of 31 patients in this series got their ears pierced after the age of ten years. This correlates well with the fact that the risk of keloids increases with each subsequent piercing, especially when the piercing is after ten years of age.[8]

From the small fraction of recurrent keloids in this series, it is evident that 6/7 recurrences were within the first year of the postoperative period.[5] Six of these patients were not operated on by Plastic Surgeons (more than half of them were operated by GPs) and were not followed regularly by the treating doctor due to various reasons. This explains the high recurrence rates during the 1^st^ postoperative year, the cause for recurrence being either improper surgical technique or irregular follow-up.[2,4]

The average duration of lesions in this study is 3.8 years and most of them had tried intralesional triamcinolone unsuccessfully (some were very hard and the drug could not be injected at all). Pressure therapy could not be applied as custom-made devices are not available here and patients are not interested in using them. So, the only way of treating them here is surgery.

It has been proven beyond doubt that surgical excision alone has very high recurrence rates ranging from 50 to 100%.[2,3,4,9] But surgical excision is a sure and predictable way of removing the lesion *in toto* and needs to be combined with other modalities. While treating keloids of exposed body parts like the ears, the aesthetic concern is as important as symptomatic improvement.

Meticulous surgical technique was used for all the cases in this series and care was taken against five proven factors (incomplete removal of keloid/scar tissue, haematoma, infection, tension on suture line and poor vascularity to wound edges) that cause undesirable scars.[4,10] The length of the wound was not taken into consideration as it does not bear any relation with the incidence of recurrence.[10]

One patient from Protocol-B, who had postoperative infection showed recurrence[5] during the first postoperative visit, *i.e.*, at about one month from the time of surgery. The other four patients who showed recurrence at the point of maximum tension over the suture line were six months from the time of surgery. Two of them belonged to the ‘recurrent keloid ‘group who were given both RTx + Intralesional triamcinolone. The other two were from the RTx group.

Recurrence in all five patients could be easily controlled with Intralesional triamcinolone. Two more patients who were operated on for keloids of the left cheek and upper sternum along with ear keloids (they were given postop RTx and subcuticular Vicryl was used for closure) showed evidence of recurrence and were controlled with Intralesional triamcinolone. The cause for this recurrence with lesions other than those of the ear in these patients is attributable to tension (four cases) over the suture line and it subsides as the scar is made supple and stretched by Intralesional triamcinolone until the tension is relaxed.

The early recurring lesions in either group were 100% susceptible to Intralesional triamcinolone therapy. But this early recurring lesion does not fulfill all clinical criteria to be diagnosed as a keloid and whether this early recurrence is a keloid or hypertrophied scar was not proven histologically. For all practical purposes, any evidence of an aberrant scar was considered as early recurrence in this study.

Comparison of the efficacy of postoperative RTx *vs.* Intralesional triamcinolone is not feasible in this study because one group of patients was given both and the RTx group had relatively more lesions which are big, complicated and with long history.

Depigmentation and atrophy of dermis were seen with all patients as a result of Intralesional triamcinolone therapy.[2,6] Pigmentation as well as atrophy were restored over a few months without any treatment.

## CONCLUSION

Surgical excision of ear keloids supplemented with radiation and/Intralesional triamcinolone with regular clinical follow-up appear to be a very reliable method with very few complications.[2–7] Meticulous surgical technique observing precautions against undesired scarring (complete removal of Keloid/scar tissue, hemostasis, asepsis, tension-free suture line and good vascularity to wound edges) is more important than the length of the scar.[10] Clinically what is most difficult to achieve is a ‘*tension-free suture line*’ which is the culprit for recurrence. Achieving a tension-free suture line is relatively more feasible with ear keloids than with lesions over many other parts of the body,[4] hence the uniformly promising results.

What appears to be most important in preventing recurrence and a new finding with this study is ‘regular clinical follow-up to encounter the early recurring lesion’, which responds in 100% of the cases to Intralesional triamcinolone in 2-3 sittings at monthly intervals.
